# Towards an individualised approach to pain management in inflammatory arthritis

**DOI:** 10.1177/1759720X261452012

**Published:** 2026-06-09

**Authors:** Zoe Rutter-Locher, Franziska Denk, Leonie S. Taams, Bruce W. Kirkham, Kirsty Bannister

**Affiliations:** Rheumatology Department, Guy’s Hospital, NHS Trust, Great Maze Pond, London SE1 9RT, UK; Department of Inflammation Biology, Faculty of Life Sciences and Medicine, School of Immunology & Microbial Sciences, King’s College University, London, UK; Wolfson Sensory, Pain and Regeneration Centre, King’s College London, London, UK; Department of Inflammation Biology, Faculty of Life Sciences and Medicine, School of Immunology & Microbial Sciences, King’s College University, London, UK; Rheumatology Department, Guy’s and St Thomas’ NHS Trust, London, UK; Department of Life Sciences, Imperial College London, South Kensington Campus, London, UK

**Keywords:** chronic pain, inflammatory arthritis, rheumatoid arthritis

## Abstract

Pain management in inflammatory arthritis (IA) remains challenging. The contribution of multiple mechanistic factors to the initiation and maintenance of pain, and the interplay of sensory, psychological and social factors that modulate perceptual outcomes, requires a multi-faceted approach that does not solely rely on inflammation-targeted therapies. Growing evidence points to the contribution of, and therefore the need to target, centrally mediated pain processes and/or peripheral sensitisation not associated with inflammatory burden. In this review, we examine current evidence on the mechanistic underpinnings of pain in both early and established IA, including novel insights regarding the manifestation of peripherally and centrally mediated pain. Further, we discuss evolving terminology used to describe pain types, including implications for clinical assessment and communication. Finally, we consider how treatment strategies – pharmacological and non-pharmacological – could fit into an individualised approach, exploring barriers to implementation in clinical practice, including limitations in pain assessment and practical challenges in service delivery.

## Introduction

Inflammatory arthritis (IA), including rheumatoid arthritis (RA), psoriatic arthritis (PsA) and spondyloarthritis (SpA), affects over half a million people in the UK and is characterised by synovial inflammation, joint destruction, disability and increased mortality.^1,2^ Advances in the management of inflammation at affected joints have led to improved disease control and data from large national surveys and clinical centre-based IA cohorts show significant reductions in swollen joint counts and inflammatory markers, alongside increased remission rates, over the last 30 years.^3,4^ For example, among patients diagnosed with RA in 2010, 59% achieved low disease activity within 5 years, compared with 32% of those diagnosed in 1990.^3^ However, no such reduction in pain outcomes (e.g. patient global assessment, quality of life and tender joint counts) has been observed over similar time periods. In fact, in some cases, pain outcomes have worsened and recent studies estimate that approximately 40% of patients with established RA continue to report persistent unacceptable pain despite treatment,^5–7^ with similar rates in PsA and axial SpA.^8,9^ The contrast between improved management of inflammation and ongoing pain highlights the need to better understand the key drivers of pain in IA.

Specific challenges in the current literature that limit our understanding of these key drivers of pain are inconsistent terminology and the absence of a framework for understanding pain heterogeneity in IA. In this narrative review, we summarise the current literature on mechanisms underpinning pain in IA, incorporating key considerations regarding terminology and proposing a framework to guide ongoing and future studies towards an individualised approach for effective pain management.

## General principles and terminology of pain

The International Association for the Study of Pain (IASP) defines pain as: ‘an unpleasant sensory and emotional experience associated with actual or potential tissue damage, or described in terms of such damage’.^10^ This definition highlights that pain, borne from a complex interaction between peripheral sensory neurons and circuits that regulate sensory processing within the spinal cord and brain, is a multi-dimensional and highly personal experience shaped by personal events, expectations and context.^11^ Acute pain serves an essential biological role by signalling tissue injury and initiating protective behaviours. However, when pain persists beyond the period of tissue repair, it has evolved into a maladaptive disease state termed ‘chronic pain’.^10^ Mechanistically, chronic pain may be underpinned by sensitisation, defined by IASP as ‘an increased responsiveness of nociceptive neurons to their normal input, and/or recruitment of a response to normally subthreshold inputs’.^10^ Clinically, sensitisation presents as hyperalgesia (exaggerated pain responses to noxious stimuli) and allodynia (pain in response to normally non-painful stimuli).^12^

When discussing pain mechanisms, consistent terminology is vital. In 2017, IASP proposed the classification of pain into three categories: nociceptive, neuropathic and nociplastic.^10^ These categories are not mutually exclusive and often coexist. Nociceptive pain arises from the activation of nociceptors by mediators released during tissue injury or inflammation. Neuropathic pain results from a disease or lesion of the somatosensory nervous system. Nociplastic pain aims to capture pain that is neither clearly nociceptive nor neuropathic in nature despite altered nociceptive function being manifest.^13^ The nociplastic pain terminology, since it provides a label for a pain type which previously lacked one, could potentially guide individualised treatment, and encourage research into the mechanisms that underlie this clinical phenotype.^13,14^ However, unlike nociceptive and neuropathic pain, nociplastic pain is a purely clinical descriptor. Indeed, the underlying biology is not known and likely involves multiple mechanisms at peripheral, spinal and supraspinal sites.^13^ Some have proposed that the mechanisms underlying nociplastic pain in IA can be summarised as: (1) ‘top down’ (dysfunction driven by maladaptivity in descending pathways, and/or genetics and/or prior experiences; these phenomena have been mechanistically assigned to so-called primary fibromyalgia) or 2) ‘bottom up’ (dysfunction driven by maladaptivity in ascending pathways; mechanistically assigned to so called secondary fibromyalgia).^15^ But even the clinical classification of fibromyalgia, one of the key conditions that aligns with the definition of nociplastic pain, is debated.^16,17^ Fibromyalgia criteria have been updated multiple times since being introduced in 1990 and they are now based on patient reported measures of widespread pain and associated symptoms.^17^ This clinical description does not include a measure of altered nociceptive function and has the potential to incorrectly stratify cohorts of patients with multiple underlying abnormalities. The designation of nociplastic pain as primary pain is problematic itself. Irritable bowel syndrome (IBS) is arguably the prototypic nociplastic pain since no peripheral aetiology is described.^13^ And yet IBS with constipation can be treated with a guanylate cyclase inhibitor, linaclotide, which acts in the gut.^18^ Fibromyalgia falls foul of similar murky ‘nociplastic pain’ interpretations – studies have shown that 50% of patients with fibromyalgia have small fibre pathology and peripheral pain-related mechanisms in fibromyalgia are evident,^19–21^ although conflicting evidence suggests that similar changes are found in numerous unrelated conditions^22^ and that histopathological findings may not translate into functional nerve impairment.^19,23^

The term nociplastic pain is also at risk of being used interchangeably with ‘central sensitisation’, although the two are conceptually very different^15^ and many argue that central sensitisation as a term has limited clinical utility and should only be used to describe a measurable physiological phenomenon. According to the IASP, central sensitisation is defined as ‘an increased responsiveness of nociceptive neurons in the central nervous system to their normal or subthreshold afferent input.’^10^ In contrast to nociplastic pain, central sensitisation refers specifically to neurophysiological processes that influence spinal and brain modulatory outputs and neuroimmunological processes.^24^ Despite this, the term central sensitisation is used in clinical settings as shorthand for ‘pain amplification’. While hyperalgesia and allodynia are symptoms of central sensitisation,^25^ the term is often also used interchangeably with fibromyalgia or chronic widespread pain, which is conceptually inaccurate.

**Figure 1.**
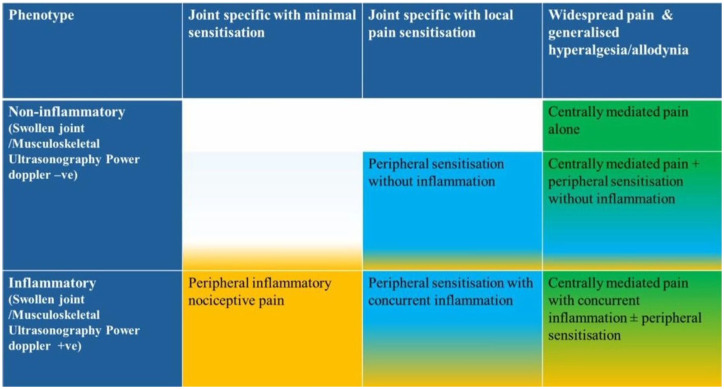
Pain phenotypes in IA. This framework illustrates different types of pain arising in IA, based on the presence or absence of active classical inflammation (clinical/US evidence) and the contribution of peripheral sensitisation and/or central mechanisms. Categories are not mutually exclusive, and overlap is common. IA, inflammatory arthritis.

As such, in this review, we have intentionally avoided the use of the terms nociplastic pain and central sensitisation, instead applying the following framework: Clinically, pain can be described according to (i) its distribution and (ii) the presence of pain sensitisation, with hyperalgesia and allodynia as key features. These features can be identified using patient-reported outcomes such as patient descriptors, validated questionnaires and fibromyalgia criteria (widespread pain index), but also more objectively using static QST (e.g. pressure pain thresholds, PPTs). Mechanistically, pain can be described as (i) peripheral nociceptive pain induced by inflammatory mediators and/or cells, (ii) pain underpinned by peripheral sensitisation either in the presence or absence of classical inflammation, (iii) centrally mediated pain in the presence or absence of classical inflammation (Figure 1). Centrally mediated pain is intentionally broad, encompassing the interplay of facilitatory and inhibitory processes at the level of the spinal cord (e.g. wind-up) and brain (e.g. pain modulation), alongside cognitive, psychological and social influences.

Bridging the gap between clinical descriptors and underlying mechanisms must therefore be approached with caution. Nevertheless, a loose framework may be helpful: widespread pain is more suggestive of centrally mediated pain, while pain sensitisation is indicative of peripheral sensitisation and/or centrally mediated processes. The key take-home message is that the relative contribution of each of these factors to the pain phenotype evolves as the disease progresses, and there is significant heterogeneity in the presentation of peripheral versus central mechanisms in patients, regardless of disease stage.

We will now summarise the evidence for different pain phenotypes and mechanisms in IA specifically, drawing on our proposed hypothetical framework.

## Mechanisms of pain in IA

Historically, pain in IA has been attributed to ongoing synovial inflammation and subsequent peripheral sensory neuron activation.^26^ The presence of ‘classical’ active inflammation is judged according to swollen joint counts and ultrasound (to include Doppler positive, i.e. vascular synovitis) and used to predict joint damage, where the therapeutic priority is to prevent irreversible loss of function.^27,28^ While residual inflammation contributes to chronic pain in some cases of IA, accumulating evidence shows that pain may persist without active classical inflammation, or may occur alongside it but be disproportionate to the inflammatory burden.^29^ This suggests a role for non-inflammatory pain-driving processes such as those driven by dysfunctional peripheral and/or central nervous system mechanisms.

**Figure 2.**
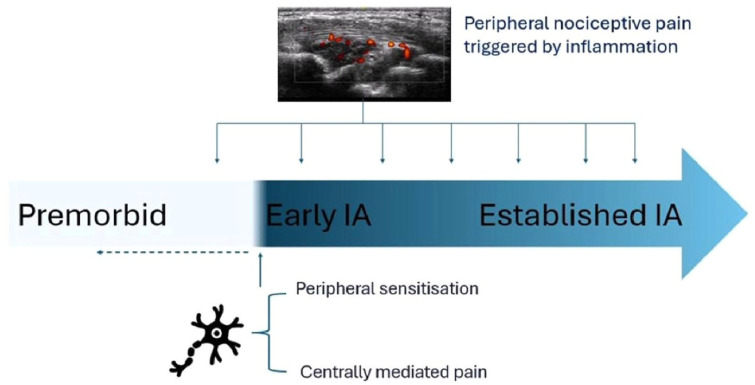
Pain mechanisms over the disease course in IA. Diagram showing a possible time course from premorbid (left) to early to established IA (right). Pain perception can occur due to a complex interplay between peripheral nociceptive mechanisms triggered by classical inflammation with peripheral sensitisation and centrally mediated pain. The prevailing assumption was that, at the onset of IA, synovial inflammation drives pain, with abnormalities in peripheral and central pain processing emerging later to contribute to persistence. However, recent evidence indicates that 20%–30% of patients already show features of centrally mediated pain at diagnosis, raising the question of whether such changes reflect pre-existing alterations in neuronal processing leading to nociception or develop because of pathological changes prior to diagnosis. Source: Based on Figure 1 in Walsh DA and McWilliams DF.^26^ IA, inflammatory arthritis.

Meanwhile, in newly diagnosed IA, pain was also largely assumed to originate from inflammation-driven peripheral nociceptive input^26^ considered necessary (at least initially) for the development of chronic pain.^30^ Indeed, for some patients, analgesic efficacy is achieved with treatments that target inflammation (i.e. intra-articular corticosteroids, NSAIDs and disease-modifying anti-rheumatic drugs). However, repeated nociceptive input can induce dysfunction in peripheral and central pain processing mechanisms,^31^ contributing to the persistence of pain as the disease progresses.^26^ This is supported by the following observations: (i) in longitudinal cohorts of RA patients, pain declines during the first 3 years after diagnosis coinciding with treatments that target inflammation before rising again,^32^ suggesting the emergence non-inflammation pain-driving mechanisms; (ii) an initial peripheral inflammatory nociceptive input precedes peripheral sensitisation and centrally mediated changes in pain processing mechanisms in IA.^11,30^ Interestingly, recent work by our group challenges the idea that such processes only emerge later in the disease course. Using detailed sensory profiling in 61 newly diagnosed IA patients, including clinical assessment, musculoskeletal ultrasound, patient-reported measures of pain sensitivity and static and dynamic quantitative sensory testing, we found that 20%–30% of individuals already showed features consistent with centrally mediated pain.^33^ Moreover, inflammatory activity explained little of the variance in pain scores, whereas central and psychosocial factors contributed substantially,^34^ suggesting that while inflammation provides an important nociceptive trigger, central and psychosocial factors may already contribute to the pain experience from the earliest stages of disease. With this in mind, in Figure 2, we update a diagram representing the time course from early to established RA that was originally proposed by Walsh and McWilliams,^26^ to reflect the contribution of dysfunctional neuronal processing from diagnosis. What remains uncertain is the extent to which these changes reflect pre-existing alterations shaped by earlier experiences, or whether they arise in response to the onset of IA itself.

### Nociceptive pain in IA

Nociceptive pain arises from the activation of peripheral sensory neurons (nociceptors) by mediators released during tissue damage or inflammation.^26,35,36^ Nociceptors are sensory neurons which respond to noxious signals^12^ comprising fibre types that may contribute to nociceptive signalling under physiological and pathophysiological conditions.^37^

In IA, a range of cells including mast cells, macrophages, neutrophils, T cells and fibroblast-like synoviocytes (FLS)^38^ release soluble mediators into the synovial fluid, which activate nociceptors. These mediators include peptides (substance P, CGRP, bradykinin), lipid mediators (prostaglandins, leukotrienes, endocannabinoids), neurotrophins, cytokines, chemokines, extracellular proteases and protons.^39^ For example, in RA, IL-1β, TNF and IL-6 are abundant in synovial fluid and in animal models can directly activate nociceptive receptors such as TRPV1.^38,40^ In PsA and axial SpA, IL-17A has also been shown to activate peripheral nociceptors.^41^ These actions contribute to pain in IA, as blockade of cytokines including TNF-α, IL-6 and IL-17A results in pain relief earlier than reductions in inflammation (as measured by swollen joint count and inflammatory markers, but not ultrasound assessment of synovial inflammation).^42–45^ JAK inhibitors, such as Baricitinib and Tofacitinib, may also have direct analgesic properties beyond their anti-inflammatory actions^46^: Human sensory neurons express JAK1 and STAT3 and are activated by STAT3-inducing cytokines enriched in RA synovial fluid, leading to STAT3 phosphorylation, induction of pain-related genes and neuronal sensitisation. These effects are blocked by the JAK inhibitor tofacitinib, supporting a direct neuronal mechanism underlying its analgesic effect.^47^

While cytokines have traditionally been studied in isolation, increasing evidence has focused on their cellular sources within the inflamed synovium.^36^ Macrophages, which produce high levels of TNF-α and IL-6, are found both in synovium and dorsal root ganglia, implicating them in pain-driving processes.^48^ FLSs release prostaglandins, neurotrophins and cytokines, all of which can lead to nociceptor sensitisation.^49,50^ The heterogeneity of synovial cellular infiltrates across patients explains, in part, inter-individual variability in disease (including pain) presentation and therapeutic response.

### Peripheral sensitisation in IA

Inflammatory mediators present during classical inflammation can rapidly induce peripheral sensitisation by directly modulating ion channels at the site of inflammation. For example, prostaglandin E2 (PGE2) phosphorylates TRPV1, increasing nociceptor excitability.^38^ However, even in the absence of classical inflammation, ultrasound findings may still evidence thickened synovial lining, indicating persistent synovial cellular infiltrates^29,51^ and recent clinical work has suggested that some patients experience peripheral sensitisation independent of active inflammation.^52^ Our group examined 158 patients with established RA and combined disease activity measures, serological markers, patient-reported outcomes and ultrasound of 44 joints to identify patient subgroups. Alongside the expected groups (inflammatory RA, fibromyalgic RA and combined RA with fibromyalgia and inflammation), we identified a fourth subgroup: patients with high pain levels but without either active inflammation or fibromyalgia. We proposed that this subgroup, accounting for 27% of the cohort, represents individuals in whom pain is driven primarily by peripheral sensitisation, a phenotype largely under-recognised in clinical practice.^52^

Mechanistically, mediators, including nerve growth factor (NGF) may contribute to peripheral sensitisation. NGF undergoes retrograde transport to the DRG, altering gene expression of pro-nociceptive molecules including TRPV1, Nav1.8 and substance P.^11^ NGF also promotes axonal sprouting, leading to hyperinnervation of joint tissues.^53^ In RA, nociceptor retraction from the synovial lining to the sublining has been observed, accompanied by an expansion of perivascular THY1+ fibroblasts.^54^ Fibroblasts themselves are emerging as key regulators of pain^55^: in vitro, activated fibroblasts increase sensory neuronal excitability,^49^ and transcriptomic studies show that pain-associated genes are enriched in synovial lining fibroblasts from RA patients with low levels of inflammation.^47,54^ Sublining fibroblasts also appear involved as they are enriched for proalgesic mediators, including leukaemia inhibitory factor and IL-11, both of which can directly activate human sensory neurons.^47^ These findings outline how, in some patients, peripheral sensitisation is maintained even when classical inflammation is suppressed.

### Centrally mediated pain in IA

Centrally mediated pain describes pain that arises from, or is maintained by, dysfunctional sensory processing in the central nervous system. It includes both spinal and supraspinal mechanisms incorporating maladaptivity in brain networks involved in emotional regulation and cognition.^11^ An imbalance in central pain modulation, shifting from inhibitory to pro-facilitatory mechanisms, can enhance pain sensitivity and contribute to persistent symptoms.^56,57^

A basis for suggesting the presence of centrally mediated pain in IA is the high prevalence of a prototypical central sensitivity syndrome, fibromyalgia, within these populations. In the general population, fibromyalgia affects fewer than 2% of individuals,^58,59^ whereas in IA, meta-analyses report much higher rates: 21% in RA, 13% in axial SpA and 18% in PsA.^60^ Fibromyalgia comorbid with IA is associated with greater pain, higher disease activity scores and poorer quality of life.^60^ Rather than a discrete comorbidity, fibromyalgia likely represents one end of a broader continuum of central nervous system dysfunction in IA.^16,61^ To capture this overlap, the concept of a ‘fibromyalgic RA’ phenotype has been proposed, defined by ⩾11 tender points.^16^ Pollard et al.^62^ showed that this phenotype could be identified using routine 28 joint counts, with a tender minus swollen joint count ⩾7. However, even this classification is limited by its binary nature, and an arguably more useful approach would be to focus on symptoms of central dysfunction such as widespread pain. Data from the large BARFOT cohort found that 34% of established RA patients reported chronic widespread pain, which was linked with higher pain levels, increased DAS28 and worse functional outcomes.^63^ Questionnaires such as the Central Sensitisation Inventory (CSI) and Pain Sensitivity Questionnaire, as well as tools developed to identify neuropathic-like pain features (e.g. painDETECT, DN4, LANSS), have been applied to IA populations. A recent meta-analysis by our group estimated a prevalence of pain sensitivity in established IA as 36% with painDETECT, 31% with DN4, 40% with LANSS and 42% with the CSI.^64^ Across instruments, pain sensitivity was consistently associated with higher pain severity, worse disease activity, increased disability, poorer quality of life and greater psychological distress.^64^ However, since these questionnaires have significant heterogeneity and limited content overlap, the generalisability and comparability of discrete questionnaire findings across studies is limited.^65^ Research is underway to develop more comprehensive and condition-specific tools. One example is the recently developed Central Aspects of Pain in Rheumatoid Arthritis questionnaire, which aims to capture a broader range of pain-related features relevant to centrally mediated pain in RA.^66^

To categorise mechanisms that underpin pain, quantitative sensory testing can be applied through the use of standardised protocols.^67^ PPTs are the minimum level of pressure required to elicit pain, with reduced thresholds at affected joints suggestive of peripheral sensitivity, and reductions at remote, non-articular sites interpreted as evidence of widespread sensitivity consistent with centrally mediated mechanisms of pain. A systematic review published in 2022 by Trouvin et al.^68^ analysed 21 studies investigating PPTs in IA, including 14 controlled studies comparing patients to healthy controls (581 patients) and 8 uncontrolled studies assessing patients only (1107 patients). In the controlled cohorts, 12 out of 14 studies found that patients with IA had lower PPTs compared to healthy controls, not only at inflamed joints but also at non-articular sites, indicating widespread pain.^68^ Findings from uncontrolled studies also showed that lower PPTs were associated with greater self-reported pain, higher tender joint counts, greater disease activity and higher levels of psychological distress.

An inferred contribution of centrally mediated mechanisms to the pain percept can also be measured according to ‘wind-up’, a pro-nociceptive phenomenon in which spinal neurons progressively increase their firing responsivity upon repeated stimulation of C-fibres above a critical rate.^69^ In human studies, a temporal summation of pain (TSP) paradigm, where repeated application of a noxious stimulus with fixed intensity leads to a progressively heightened pain response, can be used as a surrogate measure for processes that likely underpin wind-up.^70,71^ Studies have shown ‘facilitated’ (heightened) TSP in RA compared with healthy controls, but this was not replicated in larger cohort studies, including SpA cohorts.^68^ Nonetheless, facilitated TSP is consistently linked to higher pain scores, tender joint counts and disease activity.^68^ Meanwhile, conditioned pain modulation (CPM) describes a paradigm that provides a surrogate measure of processes that underpin naturally occurring pain inhibition.^72^ Most studies in IA have shown no differences in CPM between patients and healthy controls,^68^ although one suggested impaired (non-inhibitory) CPM mediated by sleep disturbance,^73^ and a recent study reported significantly smaller inhibitory CPM effects in RA and SpA.^74^ Impaired CPM has also been linked to higher pain and disease activity, and longitudinal studies suggest it may predict worse outcomes.^68^ Proinflammatory cytokines can influence central pain processing by crossing the blood-brain barrier or by signalling through afferent neural pathways; these include not only joint sensory neurons, but also the vagus nerve that carries information straight to the brainstem.^11,75^ Within the central nervous system, microglia, the resident immune cells of the brain, are thought to play a key role.^76–78^ In a recent study, patients with RA and Ankylosing spondylitis demonstrated impaired CPM at baseline compared with healthy controls. Following anti-TNF therapy, CPM responses improved, leading the authors to propose that TNF overexpression may alter descending pain inhibition in IA.^79^ However, this has not been mechanistically validated, and so these data must be interpreted with caution.

### Cortical influences on pain in IA

Neuroimaging studies in IA have identified structural and functional alterations in the brain.^11^ For example, patients with RA have increased volumes in subcortical grey matter regions such as the caudate nucleus, putamen and nucleus accumbens, which are areas implicated in the affective, cognitive and sensory-discriminative dimensions of pain.^80^ Functionally, application of painful stimuli to affected joints activates not only the thalamus and secondary somatosensory cortex, reflecting sensory discriminative processing, but also limbic structures, including the cingulate and insular cortices, which are associated with affective motivational aspects of pain processing.^44^ In RA patients with high fibromyalgia severity scores, fMRI has identified increased coupling between the default mode network and the insula, which correlates with symptom burden (including widespread pain, fatigue and tender joint counts) but not inflammation, pointing towards a centrally mediated mechanism of pain independent of inflammatory disease activity.^81,82^

Psychological distress is common in IA, contributing to reduced quality of life and impaired daily functioning.^83,84^ Meta-analyses estimate that 30%–40% of patients with RA experience depression, and there is consistent evidence linking psychological distress to greater pain severity.^84,85^ This relationship is complex and likely bidirectional: ongoing pain can trigger depression and anxiety, and psychological distress can heighten pain perception.^86^

Longitudinal studies suggest that individuals with pre-existing depression or even subthreshold symptoms of low mood are more vulnerable to developing chronic pain.^87^ In RA, depression predicts slower improvements in pain and tender joint counts.^88,89^ Similarly, higher baseline anxiety has been associated with persistently elevated pain over time.^90,91^ Interventions that reduce psychological distress improve pain outcomes via a mechanism that is proposed to involve modification of central pain processing.^92^ Depression has been linked to reduced efficacy of descending inhibitory pathways and altered activity in cortical and limbic regions involved in pain regulation.^11^ In RA, medial prefrontal cortex activity during joint palpation is greater in patients with more depressive symptoms. Psychological distress may also limit coping capacity, which is independently associated with the persistence of pain.^93^

## Treatment options for pain in IA

Pain is the primary reason patients with IA seek medical care and targeted pain management remains a major unmet clinical need.^94–96^ Chronic pain is associated with disrupted sleep, reduced physical activity and difficulties with employment^97–99^ as well as fatigue, depression and anxiety.^7,100,101^ Each of these adversely influences not only the quality of life^102^ but also treatment outcomes. In clinical practice, chronic pain can result in unnecessary escalation of immunosuppression or opioid prescribing. Despite guidelines advising against routine opioid use (NICE, EULAR), prescribing remains common, and the use of strong opioids continues to rise among individuals with IA in the UK,^103,104^ indicative of the fact that current approaches to address pain remain largely symptom- rather than mechanism-based.^94^

In arthritis in general, it is estimated that up to half of the apparent analgesic effect of treatments may be due to non-specific effects such as placebo and regression to the mean.^105^ Furthermore, analgesic responses vary between individuals, and many medications carry side effects that require personalised approaches. There are currently no comprehensive, dedicated guidelines for pain management in IA. EULAR provides guidance on non-pharmacological strategies,^34^ but full guidelines from the British Society for Rheumatology are currently in development.^106^ As a result, clinicians often rely on broader chronic pain guidelines, such as those from NICE,^107^ or they may refer to EULAR’s fibromyalgia recommendations.^108^

Treatment of pain in IA has traditionally focused on suppressing inflammatory disease activity, but evidence regarding the analgesic efficacy of individual therapies is limited, as trials rarely report pain as a specific outcome.^109^ Those who have suggested that some therapies do have superior analgesic effects.^110^ For example, JAK inhibitors such as baricitinib and upadacitinib have shown greater reductions in pain compared to adalimumab.^111^ In the RA-BEAM study, baricitinib led to greater improvements in pain scores as early as week 2, with sustained benefit through to 24 and 54 weeks.^46,112^ Similarly, upadacitinib showed greater improvements in pain compared to both adalimumab and abatacept in phase III trials.^113^ Post hoc analyses suggest that these analgesic effects are not fully explained by improvements in inflammation. In SpA, it is estimated that more than 70% of the pain relief gained from ixekizumab, an anti-IL17A, can be attributed to non-inflammatory mechanisms.^114^

Evidence for the treatment of pain in the absence of, or disproportionate to, inflammation is limited. Conventional analgesics are often used, but with varying efficacy and safety.^103^ Paracetamol has limited evidence of benefit. NSAIDs are effective in the presence of synovitis, but there is little data to support their use in non-inflammatory pain, and they carry risks such as gastrointestinal bleeding. Medications like gabapentin and pregabalin also lack evidence of benefit in IA-related pain, although pregabalin is recommended by EULAR in the management of fibromyalgia.^108^

The 2021 NICE guideline on chronic primary pain highlights the importance of shared decision-making, validation of the pain experience and development of personalised care plans.^107^ It places strong emphasis on the use of non-pharmacological strategies, with exercise showing the strongest evidence. Across 23 studies, structured physical activity, particularly aerobic exercise, reduced pain and improved quality of life both short and long term. The mechanistic underpinning of this analgesia was proposed to be activation of the descending pain inhibitory pathway(s), decreased proinflammatory cytokine response and a reduction in psychological distress.^115^ However, in fibromyalgia, exercise may lower pain thresholds and adherence remains a challenge in clinical practice.^116,117^ Psychological therapies such as Acceptance and Commitment Therapy and Cognitive Behavioural Therapy (CBT) can improve both pain and quality of life. However, the clinical effects are small. For example, a 2010 review of 27 trials reported a minimal pain reduction (effect size 0.13) over 2–14 months for CBT.^118^ Mindfulness is not recommended by NICE, but some evidence suggests it may help with pain coping and catastrophising, especially in patients with a history of depression,^92^ highlighting that different therapies may be suited to different patient groups depending on psychological profiles. Acupuncture offers short-term pain relief and improved quality of life, with effects lasting up to 3 months.^107^ Mechanistically, it is thought to engage central neurotransmitters (e.g. opioids, serotonin), but it also, peripherally, downregulates COX2 and PGE2.^119^ However, longer-term evidence is limited. In addition, EULAR recommends orthotics and a focus on sleep and weight management.^108^

Several medications have been considered for managing centrally mediated pain in IA, including amitriptyline, citalopram, duloxetine, fluoxetine, paroxetine and sertraline, based on their engagement with circuits that regulate pain modulation.^120^ Among these, duloxetine has the most robust evidence base, although no head-to-head trials have been conducted to compare effectiveness across agents.^107^ Therapies like duloxetine enhance noradrenergic transmission to push pain modulation towards inhibition.^56^ While a systematic review published in 2012 found no evidence of short-term benefit for these drugs in RA specifically,^121^ it is the case that circuits of the descending pain modulatory system, the therapeutic targets of the anti-depressants, are multiple and functionally distinct in nature. Therefore, until trials are conducted that have first identified which circuit of the descending pain modulatory system is dysfunctional in a given patient cohort, conclusions regarding the efficacy of anti-depressants for pain IA should not be drawn.

Summarising, therapeutic options are available, some of which appear to have greater analgesic efficacy. These address specific mechanistic underpinnings of pain, offering potential to feed into an individualised, mechanism-based therapeutic approach.

## Challenges to an individualised approach to pain management

**Figure 3.**
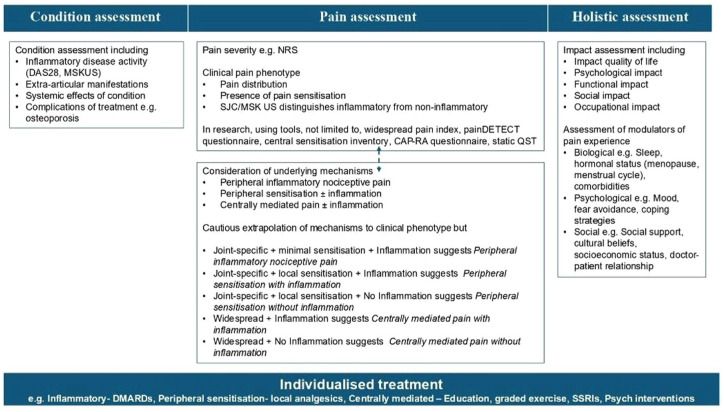
A potential framework for an individualised approach to pain treatment in IA. In this model, inflammatory disease activity and its consequences are assessed and combined with a specific assessment of pain and a holistic assessment, including condition/pain impact and modulators of the pain experience (Bio-PsychoSocial). Although clinical descriptors should not be equated directly with underlying mechanisms, features such as pain distribution and presence of sensitisation can help guide consideration of potential pain mechanisms and cautious extrapolations can be made (see also Figure 2). Joint-specific pain, minimal sensitisation and presence of classical inflammation most likely represent primarily peripheral inflammatory nociceptive pain, essentially ‘inflammation alone’, although some peripheral sensitisation is assumed to occur; in this case, treat-to-target DMARDs are the mainstay. Joint-specific pain with inflammation together with local sensitisation suggests pain from peripheral sensitisation and concurrent inflammation; here, anti-inflammatory therapy should be combined with local analgesics. Joint-specific pain without classical inflammation but with local sensitisation suggests pain from primarily peripheral sensitisation; here, local analgesics may be beneficial and escalation of DMARDs should be avoided. Widespread pain and generalised hyperalgesia/allodynia with inflammation suggests primarily centrally mediated pain with concurrent inflammation, a mixed mechanism requiring parallel treatment of both inflammatory activity and centrally mediated pain, such as education, exercise, psychological therapies or SNRIs/SSRIs as appropriate. Finally, widespread pain without inflammation is most consistent with centrally mediated pain without inflammation, which is best managed with strategies aimed at centrally mediated pain, avoiding unnecessary immunosuppression. This model highlights the importance of inflammatory disease assessment (clinical examination ± MSK US) in the management of pain in IA. However, caution is required when extrapolating symptoms to mechanisms, especially since different mechanisms often co-exist, are bi-directional, and evolve over time, meaning that assessment should be continually revisited, particularly after therapeutic changes. DMARD, disease-modifying antirheumatic drugs; IA, inflammatory arthritis.

The ability to identify a dominant ‘pain driver’ could enable more targeted and personalised pain management strategies. This concept has already been explored in other conditions, such as osteoarthritis^122^ and neuropathic pain,^123^ where clinical and sensory profiling has informed treatment selection. Recent studies have supported the feasibility of this approach in IA and have begun to identify subgroups of patients characterised by varying levels of pain severity, fatigue, psychological distress and impaired quality of life.^124,125^ There is a broad consensus that pain management in RA should be multimodal, individualised and holistic.^126^ An example of a potential framework to assess pain in IA is shown in Figure 3, highlighting the importance of identifying inflammatory disease activity alongside pain phenotype and holistic assessment.

In IA, one of the major challenges in working towards mechanism-based treatment is the ability to distinguish between pain driven by active inflammation and pain arising from peripheral and/or central nervous system dysfunction. Currently, there are no direct measures for either of these mechanisms, so the contribution of differing pain drivers must be assessed indirectly. Inflammation is often assessed clinically by examination of swollen joints, and musculoskeletal ultrasound provides a more objective measure using grey scale and power Doppler activity.^127^ Pain sensitivity can be assessed using patient-reported outcome measures, including questionnaires and quantitative sensory testing. In addition, direct patient descriptors may be useful to aid identification of inflammatory versus centrally mediated pain – a recent qualitative study by our group found that patients with features of centrally mediated pain described more overwhelming, and hard-to-define pain experiences compared to those with inflammatory pain, though both groups highlighted significant psychological, functional and social impacts.^128^ Our group also recently tested intra-articular lidocaine as a potential clinical tool to directly distinguish peripheral from centrally mediated pain, finding that patients with peripherally driven pain showed greater short-term pain relief from lidocaine compared to those with centrally mediated pain, characterised as such according to the presence of widespread hyperalgesia, fibromyalgia features and high painDETECT scores.^129^ While promising, this model requires further validation, including establishing clinical cut-offs, and future research needs to account for psychological, epigenetic and lifestyle factors that influence chronic joint pain.^130^

Chronic pain management in IA must address underlying inflammatory disease activity and its systemic consequences, such as cardiovascular disease and bone health. This added complexity makes an individualised approach especially important.^131^ For example, NSAIDs are effective for inflammatory pain but are not efficacious in non-inflammatory conditions such as fibromyalgia.^107^ In addition, identifying central pain mechanisms can help tailor treatment. For example, duloxetine may benefit those with impaired descending inhibition, but CBT or mindfulness may be more suitable for individuals with mood-related drivers of pain.^56,120^ Rheumatologists often refer the management of chronic pain to other specialties, such as pain clinics and general practitioners. However, they are uniquely positioned to lead on pain assessment in this complex patient group by complementary assessment of inflammatory disease activity alongside specific pain stratification (Figure 3). Supported by emerging guidelines and ongoing research into underlying mechanisms, rheumatologists can take ownership of pain management.^132^

There are several practical issues limiting the delivery of an individualised approach in clinical practice. A key unresolved challenge is the lack of consensus on how best to stratify patients and the heterogeneity in pain terminology. As a result, many studies have failed to stratify patients either by clinical phenotype or their likely underlying mechanisms, potentially diluting observed treatment effects.^45^ Another limitation is the lack of consensus on pain outcome measures, limiting the ability to interpret treatment efficacy from clinical trials and routine practice. A particular challenge is assessing non-pharmacological approaches, which have the strongest evidence base^107^ but whose real-world implementation is often limited by workforce capacity and inconsistent delivery. Together, these challenges contribute to an ongoing gap between the burden of chronic pain in RA, including its often-hidden wider impact on mental health and society, and the healthcare resources allocated to its management. To close this gap, future research must focus on appropriate patient stratification and the inclusion of specific pain outcomes in clinical trials.

### Limitations to consider

There are several limitations in the study design that should be considered when interpreting the evidence discussed in this review. Most studies are cross-sectional, precluding causal inference and limiting our ability to understand how pain drivers evolve over the disease course. Although this review encompasses RA, PsA and SpA, these conditions have distinct pathophysiology, and much of the literature focuses predominantly on RA. Whether findings can be extrapolated across IA subtypes remains uncertain. Finally, few studies adequately account for psychosocial factors which influence pain reporting, treatment engagement and clinical outcomes.

Another significant limitation is the absence of validated tools for measuring pain in IA. Most clinical trials of disease-modifying therapies use composite disease activity scores rather than specific pain endpoints, making it difficult to isolate analgesic efficacy. Existing questionnaires such as painDETECT, the Central Sensitisation Inventory and DN4 were not developed for IA populations and have limited content overlap, restricting comparability across studies. Across quantitative sensory testing studies, there are methodological issues including underpowering and risk of bias. Protocols for PPT assessment are highly variable, and few analyses adjust for disease duration, inflammatory activity or treatment status.^68^ In addition, while lower PPT at non-articular sites can indicate the presence of centrally mediated pain, some studies have suggested they may reflect systemic and/or genetic factors affecting peripheral nociceptor sensitivity across the body.^133^ For CPM studies, lack of protocol, outcome measure and analysis standardisation means that extracting clinically meaningful data is fraught with issues.^134–136^ Overall, findings from QST studies suggest that subsets of IA patients may experience altered pain modulation tipped towards facilitation, but linking these data to conclusions regarding the mechanistic underpinnings of pain is limited. For example, TSP and CPM protocols provide only proxy readouts of spinal facilitatory and pain modulatory processes, thus remaining limited in their ability to provide definitive mechanistic conclusions.

## Conclusion

This review provides a framework for understanding that pain in IA arises, even from diagnosis, from an interplay of peripheral nociceptive processes, peripheral sensitisation and centrally mediated mechanisms that co-exist in varying proportions across individuals and evolve over the disease course. Advancing towards individualised pain management will require improved pain stratification and clinical trials designed with pain-specific endpoints.

## Declarations

**Ethics approval and consent to participate:** Not applicable.

**Consent for publication:** Not applicable.

**Availability of data and materials:** Not applicable.
